# Analysis of latent profiles of stressors and their influencing factors in patients with breast cancer undergoing postoperative chemotherapy: a mixed-methods study

**DOI:** 10.3389/fonc.2026.1918956

**Published:** 2026-08-21

**Authors:** Jingjing Chen, Tiantian Yang, Xiaoling Yan

**Affiliations:** Department of Oncology, Affiliated Hospital of Nantong University, Nantong, Jiangsu, China

**Keywords:** breast neoplasms, influencing factors, mixed-methods research, patients undergoing chemotherapy, postoperative period, stressors

## Abstract

**Objective:**

To explore the heterogeneity of stress experiences, identify latent stressor profiles, and analyze their influencing factors in patients with breast cancer undergoing postoperative chemotherapy, in order to provide evidence for implementing precise psychosocial interventions.

**Methods:**

An explanatory sequential mixed-methods design was employed. A total of 232 patients with breast cancer undergoing postoperative chemotherapy at Nantong University Affiliated Hospital were conveniently sampled and participated in a cross-sectional survey using a general information questionnaire and the Chinese version of the Stressor Scale for Breast Cancer Patients. Latent profile analysis (LPA) was used to identify heterogeneous subgroups of stress experience, and multinomial logistic regression was conducted to analyze the influencing factors. Subsequently, 12 patients belonging to different stress profiles were purposively sampled for semi-structured interviews. Thematic analysis was performed on the interview transcripts using Colaizzi’s phenomenological method to extract core themes.

**Results:**

The stress experiences of patients with breast cancer undergoing postoperative chemotherapy presented four distinct latent profiles: ‘Low-stress Adaptive Profile’ (26.3%), ‘Moderate-High Physio-Psychological Distress Profile’ (38.4%), ‘High Medical Concerns Profile’ (20.3%), and ‘High-Stress Across Domains Profile’ (15.1%). Multinomial logistic regression analysis revealed that older age was a protective factor. For each one-year increase in age, the risk of belonging to the ‘Moderate-High Physio-Psychological Distress Profile’ (OR = 0.94, 95%CI: 0.90-0.98) and the ‘High-Stress Across Domains Profile’ (OR = 0.91, 95% CI: 0.86-0.96) decreased. Advanced clinical stage (Stage III) was an independent risk factor for belonging to the ‘High Medical Concerns Profile’(OR=2.20, 95%CI: 1.02-4.75) and the ‘High-Stress Across Domains Profile’ (OR=4.80, 95%CI: 1.85-12.45). Furthermore, low monthly family income (≤5000 RMB, OR = 3.50,95%CI: 1.25-9.80) and the presence of treatment-related complications(OR=2.80, 95%CI: 1.15-6.82)significantly increased the risk of belonging to the ‘High-Stress Across Domains Profile’. Qualitative interviews yielded three core themes: ‘Intertwined and Impactful Multiple Stressors’, ‘Depletion and Dysregulation of Internal Psychological Resources’, and the ‘Double-Edged Sword Effect of External Support Systems’.

**Conclusion:**

The stress experiences of patients with breast cancer undergoing postoperative chemotherapy exhibit significant heterogeneity, with over 70% experiencing moderate to high levels of stress. Clinical practice should incorporate stress profile identification. Targeted support, such as enhanced information and decision-making aids for patients in the ‘High Medical Concerns Profile’ and multi-dimensional integrated interventions for those in the ‘High-Stress Across Domains Profile’, is recommended to achieve precise psychosocial support.

## 1 Introduction

Breast cancer is the most common malignant tumor among women worldwide, imposing a heavy and increasing disease burden. According to the latest data from The Lancet Oncology, approximately 2.3 million new cases of female breast cancer and 764,000 deaths occurred globally in 2023 (1). In China, about 420,000 new cases were reported in 2024, making it the leading cause of cancer incidence among women. A significant trend of younger onset is also evident, with the proportion of patients aged under 40 years rising to 18% (2). Surgery followed by postoperative adjuvant chemotherapy forms the cornerstone of comprehensive treatment for breast cancer, significantly improving patient survival rates (3). However, while offering survival benefits, chemotherapy introduces a complex, multidimensional system of stressors—including physical side effects (e.g., hair loss, nausea, fatigue), body image changes, impacts on fertility, and fear of recurrence—that collectively severely erode patients’ psychological well-being and quality of life (4).Extensive domestic and international research has confirmed that patients with breast cancer undergoing postoperative chemotherapy generally experience high levels of psychological stress. One study focusing on patients during the postoperative chemotherapy period reported a positive detection rate for psychological distress as high as 97.1% (5). Another survey found that 40% of hospitalized patients receiving chemotherapy experienced moderate to severe stress levels, primarily stemming from the disease and treatment itself, drastic changes in personal life and roles, and maladjustment to the healthcare environment (6). This stress is not only strongly associated with anxiety and depression but can also undermine treatment adherence, self-efficacy, and may even impact immune function and clinical prognosis (7).

However, most existing studies adopt a variable-centered paradigm, either treating the patient population as a homogeneous whole to describe average stress levels or exploring correlations between individual stress dimensions and demographic/clinical factors (8, 9). This approach struggles to reveal heterogeneous subgroups within the patient population based on distinct patterns of stressor combinations. Clinical practice shows that some patients may be primarily distressed by body image and sexual function concerns, others more worried about family finances and career interruption, while some are deeply preoccupied with fear of recurrence and metastasis (10, 11). Overlooking this heterogeneity may lead to a “one-size-fits-all” approach to psychological intervention, lacking precision. Latent Profile Analysis (LPA), an advanced person-centered statistical method, provides a powerful tool for identifying this inherent heterogeneity. This method has been successfully applied to analyze psychological constructs in patients with breast cancer, such as psychological capital, body image, and psychological flexibility, confirming the existence of different latent classes within the patient population (12–14). Yet, there is a lack of research systematically exploring the latent profile structure of stressors specifically in patients with breast cancer undergoing postoperative chemotherapy. Identifying distinct stress pattern categories is the crucial first step towards implementing precise psychosocial assessment.

Furthermore, most existing studies on factors influencing stressors are cross-sectional surveys (15, 16). While they can reveal statistical associations, they are less capable of delving into the dynamic processes, underlying mechanisms, and personalized experiential meanings behind the formation of specific stress profiles. Stress and coping theories emphasize (17) that the stress response results from a complex interaction between an individual’s cognitive appraisal of stressful events, coping resources, and situational factors. Why do patients with similar clinical characteristics end up in different stress profiles? How do socio-cultural context, family support systems, and personal coping strategies interact in the background? These questions call for in-depth exploration through qualitative research. Therefore, to address these research gaps, this study employs an explanatory sequential mixed-methods design. First, in the quantitative phase, Latent Profile Analysis will be used, based on the validated Stressor Scale for Breast Cancer Patients (SBSCS), to cluster the stressors experienced by patients and identify their latent profile categories. Associations between demographic/clinical factors and different profiles will then be analyzed. Subsequently, in the qualitative phase, semi-structured in-depth interviews will be conducted with typical representatives from different profiles. The aim is to gain a deep understanding of the personal narratives, contextualized influencing factors, and psychological experiences that shape each stress pattern. By integrating macro-level pattern identification with micro-level experiential interpretation, this study aims to provide a solid empirical foundation for developing a tiered, precise, and personalized psychological intervention system, ultimately contributing to the improvement of overall rehabilitation and quality of life for patients with breast cancer.

## 2 Materials and methods

This study employed an explanatory sequential mixed-methods design. Quantitative research was conducted first, aiming to identify the latent profile categories of stressors and their associated factors among patients with breast cancer undergoing postoperative chemotherapy. Subsequently, based on the quantitative results, purposive sampling was used to select typical cases for qualitative research. The goal of the qualitative phase was to provide an in-depth explanation and enrichment of the quantitative findings, thereby achieving a comprehensive and profound understanding of the heterogeneity in patients’ stress experiences and its causes.

### 2.1 Quantitative study

#### 2.1.1 Participants

A convenience sampling method was used. From May 2025 to January 2026, patients with breast cancer hospitalized in the Oncology Department of Nantong University Affiliated Hospital and receiving postoperative adjuvant chemotherapy were selected as survey participants.

Inclusion criteria: (1) Diagnosed with primary breast cancer via pathological examination and having completed surgical treatment; (2) Currently receiving postoperative adjuvant chemotherapy (having completed at least one cycle); (3) Aged ≥18 years; (4) Clear consciousness, possessing basic literacy/communication skills, and able to comprehend questionnaire content; (5) Provided informed consent and voluntarily participated in the study.

Exclusion criteria: (1) Presence of severe cognitive impairment, history of mental illness, or critical illness preventing cooperation; (2) Concurrent diagnosis of other primary malignant tumors; (3) Presence of severe hearing, visual, or speech impairments affecting effective communication.

Sample size was calculated using G*Power 3.1 software. The study planned to conduct Latent Profile Analysis (LPA) and multinomial logistic regression analysis. Using multinomial logistic regression as the primary basis for calculation, parameters were set as follows: significance level α=0.05, power (1-β)=0.85, effect size (Cohen’s f^2^) set to a medium effect of 0.15, and the number of predictor variables estimated at 20. The calculation yielded a minimum required sample size of 187. Considering the possibility of invalid questionnaires (incomplete, patterned responses, etc.), the sample size was increased by 20%, resulting in a planned final sample size of 230. In this study, 240 questionnaires were distributed, and 232 valid questionnaires were recovered, with an effective response rate of 96.7%, meeting the sample size requirement. This study was approved by the Medical Ethics Committee of Nantong University Affiliated Hospital (Approval No.: 2025-K054-01). All participants provided written informed consent prior to participation.

#### 2.1.2 Measurement tools and methods

1. General Information Questionnaire.

Designed by the researchers based on a literature review, this questionnaire aimed to collect key variables potentially influencing the stress experience. It mainly included two categories: Sociodemographic Data: including age, education level, marital status, employment status, personal and family monthly income, etc. Disease and Treatment-related Data: including clinical TNM stage, surgical method, current chemotherapy cycle, and the presence of treatment-related complications.

2. Stressor Assessment Tool.

The Chinese version of the Stressor Scale for Breast Cancer Patients (SBSCS) was used for assessment (18). This scale was adapted and revised from the widely used Stressor Scale for Cancer Patients, tailored specifically for breast cancer patients. The scale comprises 25 items covering common stressors experienced by breast cancer patients during the disease and treatment process, ultimately extracting 5 core dimensions. All items are rated on a 5-point Likert scale, ranging from “No stress at all or not related to me” (1 point) to “Extreme stress” (5 points). The total score is the sum of all item scores, ranging from 25 to 125. A higher total score indicates a higher overall level of perceived stressors. The scale’s reliability and validity have been tested, demonstrating good psychometric properties. In this study, the Cronbach’s α coefficient for the total scale was 0.950, and the coefficients for the subscales ranged between 0.85 and 0.92, indicating excellent internal consistency reliability within the study sample. Its structural validity (5-factor structure) was also supported in this study by Confirmatory Factor Analysis (χ^2^/df=2.1, CFI = 0.94, TLI = 0.92, RMSEA = 0.06), making it suitable for subsequent latent profile analysis to explore patients’ heterogeneous response patterns across different stress dimensions.

#### 2.1.3 Data collection and quality control

To ensure the scientific rigor and reliability of the quantitative data, this study implemented a systematic data collection process and rigorous quality control throughout all stages. Data collection was carried out in the Oncology Department wards of Nantong University Affiliated Hospital from May 2025 to March 2026. Prior to the formal survey, the research team obtained access permission from the hospital and department, and conducted unified training for the researchers (two nurses and one head nurse) involved in data collection. This training ensured their familiarity with the study protocol, mastery of standardized communication skills, and adherence to ethical guidelines.

During the data collection phase, researchers approached patients during the inter-cycle period of chemotherapy when their physical and mental condition was relatively stable. The purpose, significance, confidentiality principles, and voluntary nature of the study were explained in detail. Questionnaires were administered only after written informed consent was obtained. Surveys were conducted in quiet, private environments. Researchers used a standardized script to provide neutral explanations of the questionnaire items. Patients completed the questionnaires independently. For participants with reading or writing difficulties, researchers read each item aloud and marked the responses on their behalf, ensuring the recorded answers accurately reflected the patients’ true intentions throughout the process.

Upon immediate retrieval of the questionnaire, the researcher checked its completeness and logical consistency, verifying and supplementing any omissions or questionable entries on the spot. In the data management stage, EpiData 3.1 software was used for double data entry by two independent personnel, followed by consistency comparison to ensure the accuracy of data transcription. Subsequently, data cleaning was performed in SPSS 26.0 software based on pre-defined criteria (e.g., missing data rate exceeding 20%, patterned responses), ultimately resulting in a clean dataset for analysis.

#### 2.1.4 Statistical methods

All quantitative data analysis for this study was performed using R language (version 4.3.1) and its relevant packages. The data processing and analysis workflow followed rigorous statistical standards. First, all data underwent cleaning and descriptive analysis. Continuous variables conforming to a normal distribution were described using mean ± standard deviation (Mean ± SD), with between-group comparisons conducted using analysis of variance (ANOVA). For those not conforming to a normal distribution, median and interquartile range were used, with between-group comparisons conducted using the Kruskal-Wallis H test. Categorical variables were described using frequency and proportion [n (%)], with between-group comparisons conducted using the chi-square test (χ^2^) or Fisher’s exact test, as appropriate. To test for common method bias, Harman’s single-factor test was performed by conducting an exploratory factor analysis (without rotation) on all scale items.

The core analysis of the study was Latent Profile Analysis (LPA), aiming to identify heterogeneous subgroups of stressor experiences among patients with breast cancer undergoing postoperative chemotherapy. Using packages such as mclustand tidyLPA, the standardized scores of the five dimensions of the Chinese version of the Stressor Scale for Breast Cancer Patients were used as manifest indicators to fit latent profile models ranging from 1 to 6 classes. The optimal number of profiles was determined by comprehensively comparing multiple model fit indices, primarily based on: 1) Information criteria, including the Akaike Information Criterion (AIC), Bayesian Information Criterion (BIC), and sample size-adjusted BIC (aBIC), with smaller values indicating better model fit; 2) Likelihood ratio tests, including the Lo-Mendell-Rubin adjusted likelihood ratio test (LMRT) and the Bootstrapped Likelihood Ratio Test (BLRT), where a p-value < 0.05 suggests that the k-class model is significantly better than the (k-1)-class model; 3) Entropy, used to evaluate classification accuracy, ranging from 0 to 1, with values closer to 1 indicating more precise classification; 4) Average posterior classification probabilities for each class, which should all be greater than 0.8; and 5) The interpretability of the model and the theoretical meaningfulness of each class profile. The final optimal model was selected by integrating statistical indices with clinical interpretability.

After determining the optimal latent profile model, individuals were classified into their most likely profile membership. Subsequently, chi-square tests, ANOVA, or non-parametric tests were used to compare differences in demographic and clinical characteristics across the different stressor profiles. Finally, to explore factors influencing an individual’s membership in a specific stressor profile, a multinomial logistic regression model (using the nnetpackage) was established. Profile membership (the class with the highest posterior probability) served as the dependent variable, and variables that were statistically significant in the univariate analyses served as independent variables. Odds ratios and their 95% confidence intervals were calculated. All statistical tests were two-tailed, with the significance level (α) set at 0.05.

### 2.2 Qualitative study

#### 2.2.1 Participants

To gain an in-depth understanding of the internal experiences and the underlying formation mechanisms associated with the different stressor profiles identified in the quantitative study, this phase employed a combined sampling strategy of purposive and maximum variation sampling. Specifically, participants were purposefully selected from each of the latent profile categories established in the quantitative phase, with the aim of including patients who could provide deep and diverse insights. Sampling sought to maximize variation across key dimensions, including stressor profile membership, age, education level, marital status, clinical stage, and treatment phase, to obtain the richest possible information. The sample size was determined by the principle of data saturation, meaning interviews continued until no new themes emerged. Ultimately, from January 2026 to March 2026, 12 patients with breast cancer receiving postoperative chemotherapy in the Oncology Department of Nantong University Affiliated Hospital were selected for semi-structured in-depth interviews. All participants were assigned codes P1–P12 to protect anonymity.

Inclusion criteria: (1) Had completed breast cancer surgery and was currently undergoing or had just completed postoperative adjuvant chemotherapy; (2) Had participated in the previous quantitative questionnaire survey and could be clearly classified into one of the stressor latent profiles; (3) Aged ≥18 years; (4) Clear consciousness, possessing good verbal expression and communication skills; (5) Provided informed consent and voluntarily participated in the interview.

Exclusion criteria: (1) Experiencing acute deterioration of their condition or having severe complications that would preclude tolerating an interview; (2) Diagnosed with a mental health disorder or cognitive dysfunction.

#### 2.2.2 Interview guide

This study employed a descriptive phenomenological research method, aiming to directly explore patients’ “lifeworld” of stress experiences. The interview guide was developed based on three key sources: (1) A review of literature related to stressors in patients with breast cancer; (2) Examination of the preliminary latent profile analysis results obtained in the quantitative phase of this study; (3) Review and feedback from an advisory panel consisting of 2 psycho-oncology specialists and 1 qualitative research methodology expert. Prior to the formal interviews, the researcher conducted pilot interviews with 2 eligible patients (who were not included in the final sample) to assess the flow, comprehensibility, and depth of the questions. Based on their feedback, adjustments were made to the phrasing and sequence of questions, resulting in the final interview guide. Core questions included: (1) “Throughout the entire process of surgery and chemotherapy, what has caused you the most stress or been the most troubling? Could you describe it in detail?”; (2) “When facing these stressors, what are the typical feelings and changes you experience physically, emotionally, or in your thoughts?”; (3) “What do you think are the reasons or circumstances that led to you feeling this kind of stress?”; (4)”How do you usually cope with or handle these stressors? How effective is that? During this process, which people, resources, or thoughts have been most helpful/least helpful to you?”; (5) “How do you view the impact of the illness and treatment on your future life (e.g., family, work, self-image)?”; (6) (Probing based on their specific stress profile) “You mentioned feeling particularly stressed about [e.g., body image/medical decision-making]. Could you talk more about your specific experiences and thoughts in this area?”

#### 2.2.3 Data collection methods

After preliminary analysis of the quantitative results, researchers contacted potential interviewees by telephone based on their assigned stress profile and basic information. The purpose, content, voluntary and confidential nature of the qualitative study, and the use of audio recording were explained in detail. Both oral and written informed consent were obtained, and an interview time was scheduled. All interviews were conducted while the patients were at the hospital for chemotherapy or follow-up appointments. Interviews took place in a private consultation room or a quiet ward room, contingent on the patient’s physical condition allowing it. Each interview was led by a researcher with a background in oncology nursing and psychological counseling, who had also received systematic training in qualitative research interviewing. Another researcher assisted by recording non-verbal information (e.g., facial expressions, pauses, emotional changes) and taking field notes. Each interview lasted approximately 20–40 minutes. The interviews were audio-recorded in their entirety. Within 24 hours of each interview, the audio recording was transcribed verbatim into text, with all personal identifying information removed. The transcript was returned to the respective interviewee for verification (member checking) to ensure the authenticity and accuracy of the content.

#### 2.2.4 Data analysis

Data were analyzed using Colaizzi’s seven-step phenomenological analysis method, facilitated by NVivo 12.0 software for data management. The specific steps were as follows: (1) Familiarization: All researchers repeatedly and carefully read all interview transcripts to gain a sense of the whole. (2) Identifying Significant Statements: Meaningful statements relevant to the research questions were extracted from the texts. (3) Formulating Meanings: The extracted significant statements were coded to formulate the meanings embedded within them. (4) Clustering Themes: The formulated meanings were aggregated, compared, and grouped based on common features to form clusters of potential themes. (5) Developing an Exhaustive Description: A detailed and thorough description of the identified themes was developed. (6) Producing the Fundamental Structure:​ The themes were refined to produce the fundamental structure that captured the essence of the experience. (7) Seeking Verification of the Findings:​ The analytical results were returned to 2–3 of the interviewees to confirm whether they accurately reflected their experiences. Final revisions were made based on their feedback. To ensure the trustworthiness of the analysis, coding was initially performed independently by two researchers. They then discussed their codes and thematic categorizations together. Any disagreements were resolved through discussion with a third, senior researcher (a nursing PhD with expertise in qualitative research) until consensus was reached.

### 2.3 Data integration and interpretation strategy for the mixed-methods study

This study employed an explanatory sequential design (19). Data integration occurred at the stages of study design and final interpretation. Firstly, the findings from the quantitative study provided a clear focus and basis for the sampling strategy and the design of the interview guide (particularly the probing questions) in the subsequent qualitative phase. Then, during the interpretation stage, a connectingapproach was adopted: the in-depth themes and mechanisms uncovered in the qualitative study were compared, cross-validated, and integrated with the profile characteristics and statistical relationships revealed by the quantitative study. In this way, the qualitative data were used to explain whydifferent stress profiles were formed, to elucidate the underlying logic behind the statistical associations, and to potentially supplement contextualized key influencing factors that were not captured by the quantitative measures. Ultimately, through this complementary and synergistic fusion of quantitative and qualitative findings, a more comprehensive, in-depth, and multidimensional understanding of the heterogeneous stressor experiences among patients with breast cancer undergoing postoperative chemotherapy​ was achieved.

## 3 Results

### 3.1 Quantitative study

#### 3.1.1 General characteristics of the participants

This study included a total of 232 breast cancer patients undergoing postoperative chemotherapy. The age of the patients ranged from 28 to 70 years, with a mean age of (48.6 ± 9.2) years. 230 (99.1%) participants were female. Detailed sociodemographic and clinicopathological characteristics of the participants are presented in **Table 1**.

**Table 1:** General characteristics patients with breast cancer undergoing postoperative chemotherapy (N = 232).

Characteristic	Category	n	(%)/(Mean ± SD)
Age (years)		232	48.6 ± 9.2
Gender	Female	230	99.1
	Male	2	0.9
Marital Status	Married	205	88.4
	Unmarried/Divorced/Widowed	27	11.6
Education Level	Junior high school or below	72	31.0
	High school/Technical secondary school	83	35.8
	College/Associate degree	77	33.2
Employment Status	Employed	113	48.7
	Unemployed/Left job	47	20.3
	Retired	72	31.0
Monthly Family Income (Yuan)	≤5000	61	26.3
	5001~10000	90	38.8
	≥10000	81	34.9
Clinical Stage	Stage I	33	14.2
	Stage II	102	44.0
	Stage III	97	41.8
	Stage IV	0	0.0
Surgical Method	Breast-conserving surgery	68	29.3
	Modified radical mastectomy	142	61.2
	Other	22	9.5
Current Chemotherapy Cycle		232	3.8 ± 2.1
Treatment-related Complications	Yes	167	72.0
	No	65	28.0

#### 3.1.2 Latent profile analysis of stressors in patients with breast cancer undergoing postoperative chemotherapy

To identify potential heterogeneous subgroups of stressor experiences among patients with breast cancer undergoing postoperative chemotherapy, latent profile analysis (LPA) was conducted on 232 patients using the standardized scores of the five dimensions of the stressor scale as indicator variables. Competing models with one to five latent profiles were fitted sequentially, and their fit indices are presented in **Table 2**. Model selection was based on a comprehensive evaluation of statistical indicators, model parsimony, and clinical interpretability. As shown in **Table 2**, the AIC, BIC, and aBIC values decreased progressively with an increasing number of profiles, indicating gradual improvement in model fit. The LMRT and BLRT results suggested that the four-profile model was statistically superior to the three-profile model (p < 0.05), whereas the five-profile model did not show significant improvement over the four-profile model (LMRT p = 0.214). Furthermore, the four-profile model yielded the highest entropy value (0.891), indicating optimal classification accuracy, and the average posterior probabilities for all profiles exceeded 0.85, demonstrating good model stability. The five-profile model contained a small class (8.6%) with poor interpretability. Therefore, the four-profile model was selected as the optimal model.

**Table 2:** Model fit indices for the latent profile analysis of stressors in postoperative postoperative patients with breast cancer (N = 232).

Model	AIC	BIC	aBIC	Entropy	LMRT (p)	BLRT (p)	Class proportions (%)
1-Profile	4523.18	4555.24	4529.15	—	—	—	100.0
2-Profile	4289.77	4342.86	4303.71	0.843	<0.001	<0.001	35.8/64.2
3-Profile	4188.45	4262.58	4200.28	0.862	0.027	<0.001	28.0/41.4/30.6
**4-Profile**	**4123.01**	**4218.17**	**4152.90**	**0.891**	**0.038**	**<0.001**	**26.3/38.4/20.3/15.1**
5-Profile	4098.34	4214.53	4136.20	0.875	0.214	0.103	8.6/31.9/25.9/20.3/13.4

AIC, Akaike Information Criterion; BIC, Bayesian Information Criterion; aBIC, sample-size adjusted BIC; LMRT, Lo-Mendell-Rubin adjusted likelihood ratio test; BLRT, Bootstrap likelihood ratio test. The row in bold indicates the selected optimal model. The class proportions for the 4-profile model are, in order: Profile 1 (26.3%), Profile 2 (38.4%), Profile 3 (20.3%), Profile 4 (15.1%).

To further evaluate the robustness of model selection, a sensitivity analysis was conducted on the three-profile model. The fit indices for the three-profile model were: AIC = 4188.45, BIC = 4262.58, aBIC = 4203.71, entropy = 0.843, LMRT p < 0.001, BLRT p < 0.001. The proportions of the three profiles were: Profile 1 (28.0%, n = 65), Profile 2 (41.4%, n = 96), and Profile 3 (30.6%, n = 71), indicating a more balanced class distribution compared with the four-profile model.

**Figure 1:**
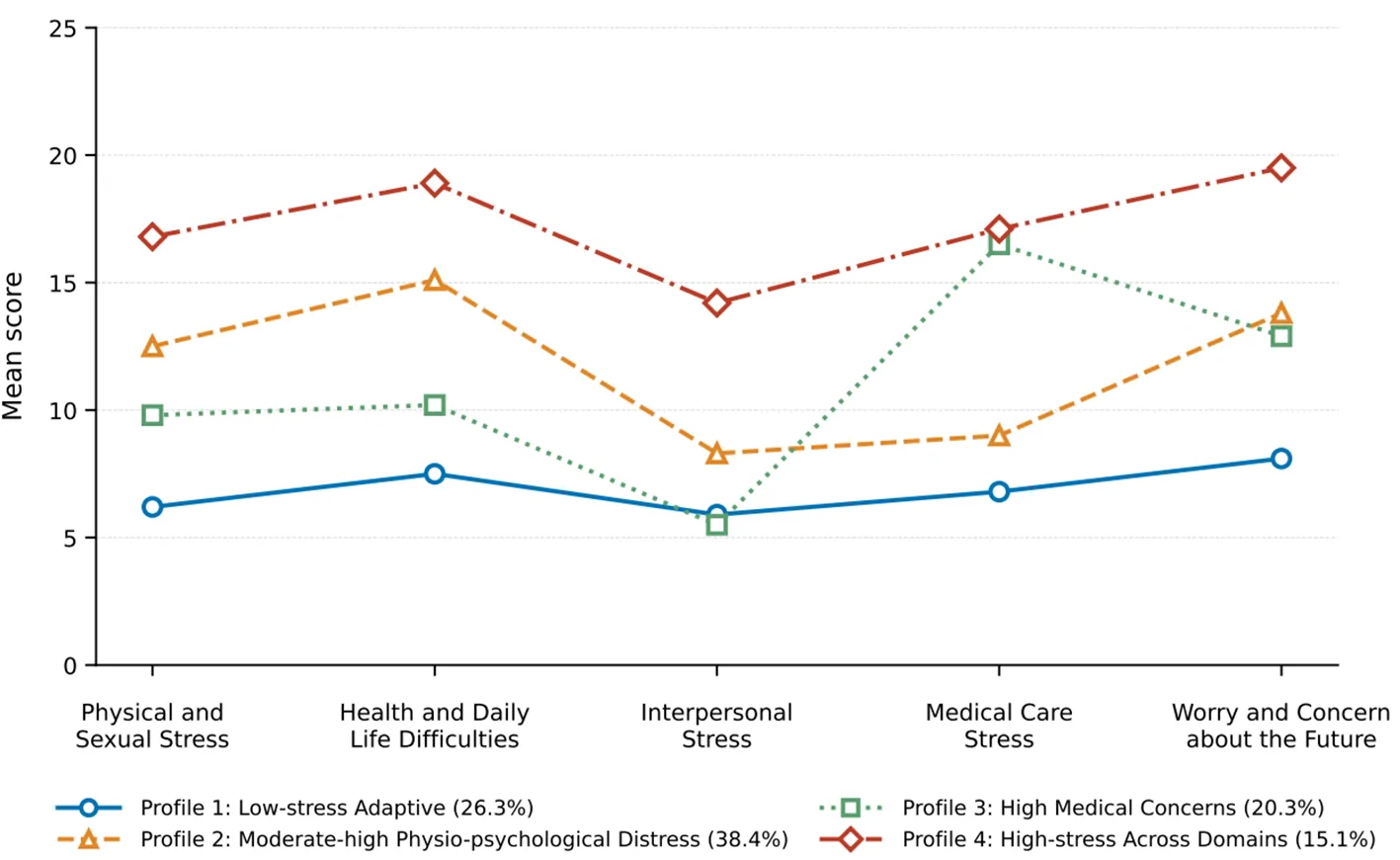
Profile plot of standardized scores for the four latent profiles of stressors in patients with breast cancer undergoing postoperative chemotherapy.

The patterns of standardized scores across the five stressor dimensions in the three-profile model (see **Supplementary Figure 1**) were as follows: Profile 1 had the lowest overall scores across all five dimensions and was labeled “Low-stress Adaptive”; Profile 2 showed moderately high scores on Physical and Sexual Stress, Health and Daily Life Difficulties, and Worry and Concern about the Future, but lower scores on Interpersonal Stress and Medical Care Stress, and was labeled “Moderate-high Physio-psychological Distress”; Profile 3 had the highest scores on all dimensions and was labeled “High-stress.” Compared with the four-profile model, the three-profile model merged the “High Medical Concerns” and “High-stress Across Domains” profiles from the four-profile model into a broader “High-stress” profile, thereby losing the fine-grained distinction of medical-related concerns as a unique dimension. However, from a clinical practicality perspective, the three-profile model provided more balanced subgroup sample sizes, facilitating subsequent implementation of tiered interventions. Both models have their respective advantages: the four-profile model demonstrated superior statistical fit (higher entropy, significant LMRT) and was able to identify the specific stress pattern of medical concerns; the three-profile model offered a simpler structure with larger subgroup sizes, potentially more suitable for application in resource-limited clinical settings.Based on the standardized score patterns of the four latent profiles across the five stressor dimensions (see **Figure 1**, **Table 3**), they were named and described as follows:

**Table 3:** Comparison of different latent profiles on stressor dimensions and total score (Mean ± SD).

Latent profile	Physical and sexual stress	Health and daily life difficulties	Interpersonal stress	Medical care stress	Worry and concern about the future	Total stressor score
Profile 1: Low-stress Adaptive (n=61)	6.2 ± 1.8	7.5 ± 2.0	5.9 ± 1.7	6.8 ± 1.9	8.1 ± 2.2	34.5 ± 5.2
Profile 2: Moderate-high Physio-psychological Distress (n=89)	12.5 ± 2.1	15.1 ± 2.9	8.3 ± 2.0	9.0 ± 2.2	13.8± 2.7	58.7 ± 6.8
Profile 3: High Medical Concerns (n=47)	9.8 ± 2.3	10.2 ± 2.5	5.5 ± 1.5	16.5 ± 2.7	12.9 ± 2.5	54.9 ± 6.1
Profile 4: High-stress Across Domains (n=35)	16.8 ± 2.5	18.9 ± 3.0	14.2 ± 2.8	17.1 ± 2.9	19.5 ± 3.1	86.5 ± 8.3
F-value	185.32	201.47	176.58	245.63	189.74	312.89
P-value	<0.001	<0.001	<0.001	<0.001	<0.001	<0.001
*Post-hoc* comparison (LSD)	4>2>3>1	4>2>3>1	4>2>1,3	3,4>2>1	4>2,3>1	4>2,3>1

(1) Low-stress Adaptive (26.3%): Scored lowest on all dimensions. (2) Moderate-high Physio-psychological Distress (38.4%): Scored relatively high on Physical and Sexual Stress, Health and Daily Life Difficulties, and Worry about the Future. (3) High Medical Concerns (20.3%): Scored significantly highest on Medical Care Stress and lowest on Interpersonal Stress. (4) High-stress Across Domains (15.1%): Scored highest on all dimensions.

Profile 1: Low-stress Adaptive Profile (26.3%): This profile comprised 61 patients. Its defining characteristic was the lowest scores across all five stressor dimensions, which were significantly lower than those of the other profiles (p < 0.001). This indicates that this group of patients perceived the lowest overall level of stress throughout the treatment process, demonstrating a state of better psychological adaptation.

Profile 2: Moderate-high Physio-psychological Distress Profile (38.4%): This was the largest subgroup, comprising 89 patients. Its stress pattern was characterized by moderately high scores on the three dimensions of “Physical and Sexual Stress,” “Health and Daily Life Difficulties,” and “Worry and Concern about the Future,” while scores on “Interpersonal Stress” and “Medical Care Stress” were relatively lower. This suggests that the core stressors for this group primarily originated from concerns about their own body image, functional status, symptom burden, and anxiety regarding the disease prognosis.

Profile 3: High Medical Concerns Profile (20.3%): This profile comprised 47 patients. Its most prominent feature was a significantly higher score on the “Medical Care Stress” dimension compared to all other profiles (p < 0.001), coupled with a relatively high score on “Worry and Concern about the Future.” Conversely, it had the lowest score on the “Interpersonal Stress” dimension. This indicates that the stress experience for this group was highly concentrated on confusion and worries regarding treatment decisions, doctor-patient communication, medical information, costs, and the healthcare system itself.

Profile 4: High-stress Across Domains Profile (15.1%): This profile comprised 35 patients. Its stress pattern was characterized by the highest level of scores reported across all five dimensions, which were significantly higher than those of the other three profiles (p < 0.001). This group of patients endured comprehensive, high-intensity stress from physiological, psychological, social, and healthcare system domains.

#### 3.1.3 Results of univariate analysis of differences in demographic and clinical characteristics across stressor profiles

The univariate analysis results (see **Table 4**) indicated that the different stressor profiles showed statistically significant differences in terms of age, monthly family income, clinical stage, current chemotherapy cycle, and the presence of treatment-related complications (p < 0.05).

**Table 4:** Univariate analysis of demographic and clinical characteristics across different stressor profiles [n(%), x̄ ± s].

Characteristic	Profile 1: (n=61)	Profile 2: (n=89)	Profile 3: (n=47)	Profile 4: (n=35)	Statistic	P
Age (years)	52.3 ± 8.1	48.1 ± 9.0	47.5 ± 9.5	44.7 ± 8.9	5.871^a^	<0.001
Gender					0.607^b^	0.895
Female	60 (98.4)	88 (98.9)	47 (100.0)	35 (100.0)		
Male	1 (1.6)	1 (1.1)	0 (0.0)	0 (0.0)		
Marital Status					1.702^c^	0.636
Married	57 (93.4)	77 (86.5)	42 (89.4)	29 (82.9)		
Unmarried/Divorced/Widowed	4 (6.6)	12 (13.5)	5 (10.6)	6 (17.1)		
Education Level					5.223^c^	0.516
Junior high or below	15 (24.6)	30 (33.7)	15 (31.9)	12 (34.3)		
High school/Technical sec.	25 (41.0)	30 (33.7)	18 (38.3)	10 (28.6)		
College/Associate degree	21 (34.4)	29 (32.6)	14 (29.8)	13 (37.1)		
Employment Status​					4.321^c^	0.633
Employed	25 (41.0)	45 (50.6)	24 (51.1)	19 (54.3)		
Unemployed/Left job	9 (14.8)	19 (21.3)	9 (19.1)	10 (28.6)		
Retired	27 (44.3)	25 (28.1)	14 (29.8)	6 (17.1)		
Monthly Family Income (Yuan)					25.731^c^	<0.001
≤5000	10 (16.4)	20 (22.5)	15 (31.9)	16 (45.7)		
5001-10000	22 (36.1)	38 (42.7)	20 (42.6)	10 (28.6)		
>10000	29 (47.5)	31 (34.8)	12 (25.5)	9 (25.7)		
Clinical Stage					18.942^c^	0.004
Stage I-II	36 (59.0)	50 (56.2)	23 (48.9)	8 (22.9)		
Stage III	25 (41.0)	39 (43.8)	24 (51.1)	27 (77.1)		
Surgical Method					2.114^c^	0.909
Breast-conserving	21 (34.4)	26 (29.2)	13 (27.7)	8 (22.9)		
Modified radical mastectomy	35 (57.4)	54 (60.7)	30 (63.8)	23 (65.7)		
Other	5 (8.2)	9 (10.1)	4 (8.5)	4 (11.4)		
Current Chemotherapy Cycle	3.1 ± 1.8	3.9 ± 2.0	4.3 ± 2.2	4.9 ± 2.3	4.325^a^	0.006
Treatment-related Complications					17.224^c^	0.001
Yes	35 (57.4)	66 (74.2)	36 (76.6)	30 (85.7)		
No	26 (42.6)	23 (25.8)	11 (23.4)	5 (14.3)		

^a^F-value (one-way ANOVA).

^b^Fisher’s exact test probability value; cχ^2^ value

#### 3.1.4 Analysis of influencing factors for stressor profiles in patients with breast cancer undergoing postoperative chemotherapy

To identify the independent factors associated with patients’ membership in specific stressor profiles, all candidate variables—age, monthly family income, clinical stage, current number of chemotherapy cycles, and presence of treatment-related complications were simultaneously entered into a multinomial logistic regression model, with the “Low-stress Adaptive” profile (the least stressful profile) serving as the reference group. This analysis was conducted on the basis of variables that showed statistical significance in univariate analysis, in order to adjust for the mutual confounding effects among the factors. In the multinomial logistic regression, the categorical variables were coded as follows: Dependent variable (stressor profile):​1 = Low-stress Adaptive (reference), 2 = Moderate-high Physio-psychological Distress, 3 = High Medical Concerns, 4 = High-stress Across Domains. Independent variables:​ Monthly family income (1 = ≤5000 Yuan, 2 = 5001–10000 Yuan, 3 = >10000 Yuan [reference]), Clinical stage (0 = Stage I-II [reference], 1 = Stage III), Presence of treatment-related complications (0 = No [reference], 1 = Yes). Continuous variables (age, current chemotherapy cycle) were entered as raw values.

**Figure 2:**
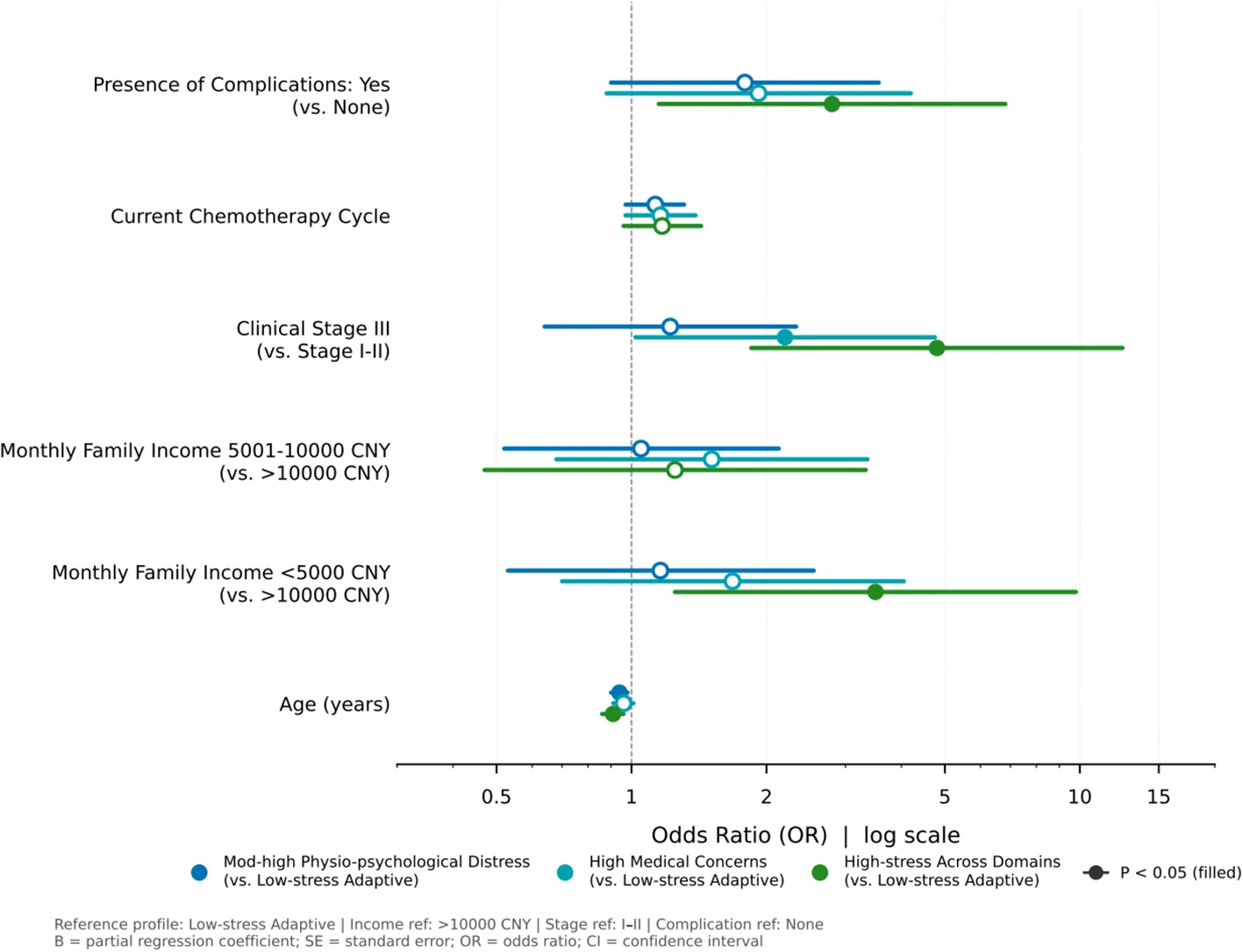
Forest plot of the multinomial logistic regression analysis for factors influencing stressor profiles in patients with breast cancer undergoing postoperative chemotherapy.

Older age was a significant protective factor. For each one-year increase in age, the risk of belonging to the “Moderate-high Physio-psychological Distress” profile (OR = 0.94, 95% CI: 0.90-0.98) and the “High-stress Across Domains” profile (OR = 0.91, 95% CI: 0.86-0.96) decreased by 6% and 9%, respectively. Monthly family income showed a gradient effect. Compared to patients with a high income (>10000), those with a low income (≤5000) had a 3.5 times higher risk of belonging to the “High-stress Across Domains” profile (OR = 3.50, 95% CI: 1.25-9.80). Compared to patients with Stage I-II disease, those with Stage III disease had a significantly increased risk of belonging to the “High Medical Concerns” profile (OR = 2.20, 95% CI: 1.02-4.75) and the “High-stress Across Domains” profile (OR = 4.80, 95% CI: 1.85-12.45). The presence of treatment-related complications also increased the risk of belonging to the “High-stress Across Domains” profile by 2.8 times (OR = 2.80, 95% CI: 1.15-6.82). The number of current chemotherapy cycles did not show an independent effect after controlling for other factors (P > 0.05). See **Table 5** and **Figure 2**.

**Table 5:** Multinomial logistic regression analysis of factors influencing stressor profiles in patients with breast cancer undergoing postoperative chemotherapy.

Variable	Profile vs. reference	B	SE	Wald χ^2^	OR (95% CI)	P-value
Age (years)						
	Mod-high Physio-psych Distress	-0.06	0.02	7.84	0.94 (0.90-0.98)	0.005
	High Medical Concerns	-0.04	0.03	2.13	0.96 (0.91-1.01)	0.144
	High-stress Across Domains	-0.10	0.03	10.12	0.91 (0.86-0.96)	0.001
Monthly Family Income (Yuan)						
<5000	Mod-high Physio-psych Distress	0.15	0.40	0.14	1.16 (0.53-2.55)	0.708
	High Medical Concerns	0.52	0.45	1.34	1.68 (0.70-4.05)	0.247
	High-stress Across Domains	1.25	0.52	5.76	3.50 (1.25-9.80)	0.016
5001-10000	Mod-high Physio-psych Distress	0.05	0.36	0.02	1.05 (0.52-2.13)	0.889
	High Medical Concerns	0.41	0.41	1.00	1.51 (0.68-3.36)	0.317
	High-stress Across Domains	0.22	0.50	0.19	1.25 (0.47-3.33)	0.662
Clinical Stage						
Stage III	Mod-high Physio-psych Distress	0.20	0.33	0.36	1.22 (0.64-2.33)	0.548
	High Medical Concerns	0.79	0.39	4.10	2.20 (1.02-4.75)	0.043
	High-stress Across Domains	1.57	0.45	12.18	4.80 (1.85-12.45)	<0.001
Current Chemotherapy Cycle						
	Mod-high Physio-psych Distress	0.12	0.08	2.25	1.13 (0.97-1.31)	0.134
	High Medical Concerns	0.15	0.09	2.78	1.16 (0.97-1.39)	0.095
	High-stress Across Domains	0.16	0.10	2.56	1.17 (0.96-1.43)	0.110
Presence of Complications						
Yes	Mod-high Physio-psych Distress	0.58	0.35	2.74	1.79 (0.90-3.56)	0.098
	High Medical Concerns	0.65	0.40	2.64	1.92 (0.88-4.20)	0.104
	High-stress Across Domains	1.03	0.45	5.24	2.80 (1.15-6.82)	0.022

Partial regression coefficient; SE, standard error; OR, odds ratio; CI, confidence interval. The reference category for the dependent variable was the “Low-stress Adaptive” profile. The reference categories for the independent variables were: monthly family income >10,000 RMB, clinical stage I–II, and absence of treatment-related complications. Age and current number of chemotherapy cycles were entered as continuous variables. All ORs were derived from a multivariable logistic regression model that included all independent variables listed in the table simultaneously.

Results of the sensitivity analysis showed that after categorizing age into tertiles (≤45 years, 46–59 years, ≥60 years), the direction of associations between age groups and stress profile membership was consistent with that of the continuous variable analysis. Moreover, the protective effect was more pronounced in the ≥60 years age group (OR = 0.42, 95% CI: 0.20–0.88).

### 3.2 Qualitative findings

This phase of the study was based on in-depth interviews with 12 patients with breast cancer undergoing postoperative chemotherapy, who were purposively sampled from the distinct stressor profiles identified in the quantitative analysis. Rich narrative data were collected, totaling 312 minutes of interview time, which were transcribed into approximately 58,000 words of text. The 12 interviewees in this study were evenly recruited from the four stress profiles (three per profile). Their ages ranged from 32 to 61 years, as shown in **Table 6**. Through systematic coding and thematic analysis of the textual data, three core themes and seven subthemes were extracted. This analysis aimed to reveal, from the patients’ own perspectives, the profound meaning, multidimensional sources, and coping processes inherent in their stress experiences.

**Table 6:** Characteristics of qualitative interview participants and distribution across profiles (n=12).

Participant ID	Stress profile	Age (years)	Clinical stage	Monthly family income(RMB)	Complications
P1	High Medical Concerns	47	Stage III	≤5,000	Yes
P2	Moderate-high Physio-psychological Distress	52	Stage II	5,001–10,000	No
P3	Low-stress Adaptive	58	Stage I	>10,000	No
P4	High Medical Concerns	45	Stage III	5,001–10,000	Yes
P5	Low-stress Adaptive	61	Stage I	>10,000	No
P6	High-stress Across Domains	38	Stage III	≤5,000	Yes
P7	High Medical Concerns	49	Stage II	5,001–10,000	No
P8	High-stress Across Domains	35	Stage III	≤5,000	Yes
P9	Moderate-high Physio-psychological Distress	44	Stage II	5,001–10,000	No
P10	Low-stress Adaptive	55	Stage I	>10,000	No
P11	High-stress Across Domains	32	Stage III	≤5,000	Yes
P12	Moderate-high Physio-psychological Distress	50	Stage II	5,001–10,000	No

#### 3.2.1 Theme 1: the intertwined nature of multidimensional stressors

Patients’ stress was not singular or isolated. It originated from challenges across multiple domains—physical, role-related, and existential. These stressors were interwoven and often reinforced one another, creating a composite and complex stress experience.

Subtheme 1.1: Disruption of Body Image and Loss of Functionality.

Significant bodily changes resulting from surgery and chemotherapy—such as hair loss, surgical scars, breast absence or deformation, and persistent fatigue—constituted a core stressor for all patients. This was not merely a physical alteration but profoundly challenged their feminine identity and sense of bodily autonomy. “Looking at my bald self in the mirror, with that long scar on my chest, I really don’t recognize myself. This isn’t me anymore. It feels like my body has been ‘ruined’—it feels alien and shameful.”(P6, High-stress Across Domains Profile).

Subtheme 1.2: Disruption of Social Roles and an Associated Identity Crisis.

The illness forcibly removed patients from, or severely limited their functioning in, their pre-diagnosis occupational and family roles. This sudden shift triggered intense feelings of worthlessness and uncertainty about the future. “After getting sick, I had to stop working and couldn’t manage household duties anymore. I used to be the pillar of my family, but now I feel like the biggest burden. I feel useless, like I contribute nothing, and my heart feels utterly empty.”(P8, High-stress Across Domains Profile).

Subtheme 1.3: Existential Anxiety Fueled by Prognostic Uncertainty.

Fear of cancer recurrence, metastasis, and death permeated the entire treatment trajectory. This pervasive sense of uncertainty and lack of control over the future formed a constant backdrop of stress. “Every follow-up appointment feels like awaiting a verdict. The slightest bodily discomfort makes me wonder if it’s a metastasis. The most tormenting part isn’t the present suffering, but that constant, nagging anxiety about the future—not knowing what tomorrow holds.”(P2, Moderate-high Physio-psychological Distress Profile).

#### 3.2.2 Theme 2: depletion of internal resources and impaired self-regulation

Confronted with external stressors, patients’ internal psychological regulation processes were highly challenging. Emotional distress, cognitive burdens, and self-doubt compounded each other, leading to the ongoing depletion of their psychological resources.

Subtheme 2.1: Emotional Overload and Vicious Cycles of Distress.

Patients commonly grappled with a surge of complex emotions, including anxiety, depression, and sorrow. Under high stress, negative emotions and physical sensations could reinforce each other, easily trapping them in a vicious cycle. “I break down so easily now; the smallest thing makes me want to cry. Then, the more I cry, the more useless I feel, the more hopeless the future seems, and I feel even more physically exhausted. It’s a complete, inescapable vicious cycle.”(P11, High-stress Across Domains Profile).

Subtheme 2.2: Decision-Making Fatigue and Information Overload.

Particularly for patients characterized as the “High Medical Concerns” profile, the necessity to make continual medical decisions amidst complex treatment options and often contradictory information (especially from online sources) resulted in significant cognitive burden and decision fatigue. “Which surgery option? Domestic or imported chemotherapy drugs? Every choice feels like a matter of life and death. I scoured for information everywhere, asked different people, and my mind was in complete chaos. In the end, I was utterly drained, simply unable to make any more decisions.”(P4, High Medical Concerns Profile).

Subtheme 2.3: Disease-Related Stigma and Subsequent Social Withdrawal.

Some patients, especially younger ones, experienced intense shame associated with the “cancer patient” label. Fearing discrimination or pity, they proactively withdrew from social interactions, which in turn exacerbated their feelings of loneliness. “I don’t want my friends and colleagues to see me like this, nor do I want them talking to me in that sympathetic tone. So, I’ve left all the group chats, shut myself away. But doing that only makes me feel lonelier.”(P8, High-stress Across Domains Profile).

#### 3.2.3 Theme 3: the dual-potential of external supports: buffers versus stressors

External resources such as family, the healthcare system, and financial means are theoretically key buffers against stress. In reality, however, they exhibited a complex “double-edged sword” effect, potentially alleviating stress but also risked becoming novel sources of distress.

Subtheme 3.1: The Burden of Gratitude: When Family Support Evokes Guilt.

While the caregiving and emotional support from family were invaluable, some patients paradoxically developed strong feelings of guilt and perceived themselves as a burden because of it. “My husband’s work has suffered because he takes care of me. My parents are old and still worry so much about me. The more grateful I am to them, the more I resent myself, feeling like I’m dragging the whole family down.”(P1, High Medical Concerns Profile).

Subtheme 3.2: The Critical Role of Clinical Communication: Fostering Trust or Exacerbating Distress.

The professional competence and, crucially, the communication style of healthcare providers directly shaped patients’ “medical-related stress.” Compassionate, clear, and informative communication greatly alleviated anxiety, whereas rushed, vague, or paternalistic interactions amplified feelings of uncertainty and helplessness. “My attending doctor always takes a moment during rounds to say a few extra words, gives me a pat, explains whywe’re doing this step of the treatment. That makes me feel so secure, like I have solid support. But some doctors just say ‘follow the plan,’ and then I’m left feeling terribly uncertain and panicky inside.”(P7, High Medical Concerns Profile).

Subtheme 3.3: The Tangible and Enduring Burden of Financial Toxicity.

The direct costs of treatment, severe income reduction due to work interruption, and deep concerns about the family’s long-term financial stability constituted a heavy, concrete form of pressure. This was especially acute for younger patients and those with lower incomes. “The monthly bills for medication and check-ups are like a mountain. My job is on hold; I only get a basic income now. What scares me most is the thought of the money running out before I’m cured. That would be true despair. I don’t dare to think about the future.”(P11, High-stress Across Domains Profile).

### 3.3 Integrated results of the mixed-methods study

Integrating the quantitative and qualitative findings revealed a relationship of mutual verification, elaboration, and complementarity between the two strands in elucidating the characteristics, influencing factors, and formation mechanisms of the stressor profiles among patients with breast cancer undergoing postoperative chemotherapy. Together, they constructed a more comprehensive and multidimensional understanding of the heterogeneity in this population’s stress experiences. The specific integrative analysis is presented in **Table 7**.

**Table 7:** Integration of mixed-methods results.

Quantitative findings	Qualitative findings	Integrated interpretation
Four heterogeneous pressure profiles were identified.	Revealed distinct core stress foci and psychological experiences among patients from different profiles. For instance, patients in the ‘High Medical Concerns’ profile elaborated on their confusion regarding treatment decisions and their sensitivity to communication details, while those in the ‘High-stress Across Domains’ profile expressed a sense of being overwhelmed.	Confirmation and Concretization. The qualitative descriptions provided concrete real-world illustrations and exemplars for the abstract profile types identified statistically, making the profile characteristics tangible and relatable.
Older age was a protective factor against high-stress profiles.	Younger patients commonly expressed more intense experiences of social role disruption, financial pressure, loss of control over the future, and disease-related stigma.	Convergence. The qualitative data provided a mechanistic explanation for the statistical association that “older age is protective”: younger patients face heavier social expectations and practical burdens, leading to more intense stress reactions.
Advanced clinical stage and the presence of treatment-related complications were significant risk factors for belonging to the ‘High-stress Across Domains’ profile.	Patients with advanced disease and multiple complications described an experience of being “comprehensively besieged” by physical discomfort, treatment side effects, and profound worry about the future, feeling unable to cope.	Confirmation and Pathway Elucidation. The qualitative data supported the association between these clinical factors and high-stress experience, and further suggested that these factors may catalyze the accumulation of overall stress by exacerbating patients’ physical suffering and sense of illness uncertainty.
Lower monthly family income was a risk factor for belonging to the ‘High-stress Across Domains’ profile.	Patients with lower incomes provided detailed accounts of the direct anxiety caused by treatment costs, the guilt stemming from an inability to work and support the family, and worries about the family’s long-term financial situation.	Confirmation and Enrichment. The qualitative study not only validated the importance of the economic factor but also enriched its meaning, revealing that financial pressure is tightly interwoven with psychological-emotional aspects like “guilt” and “family responsibility,” jointly exacerbating the patient’s psychological burden.
The ‘High Medical Concerns’ profile scored significantly higher on the ‘Medical Care Stress’ scale dimension.	Patients belonging to this profile frequently mentioned feeling confused by complex treatment information, experiencing great pressure when needing to make medical choices, and paying close attention to the detail and attitude of doctors’ communication.	Confirmation and Focus Clarification. The qualitative data clearly indicated that the high scores for this profile did not reflect generalized worry; rather, the core focus was on the uncertainty and low sense of control regarding medical information and the decision-making process, providing a key to understanding the essence of this profile.
(The stressor scale did not directly measure this)	Multiple patients, especially younger ones, voluntarily mentioned the shame (stigma) arising from the “cancer patient” label and the consequent social avoidance behaviors.	Extension and Supplementation. The qualitative study identified and emphasized “disease-related stigma” and “social withdrawal” as an independent and significant psychosocial stressor for breast cancer patients, a dimension not fully captured by the existing standardized scale.
(The stressor scale measured ‘Medical Care Stress’ but at a macro level)	Patients repeatedly emphasized that details in healthcare providers’ communication—such as tone, patience, and whether encouragement was given—greatly influenced their sense of security and anxiety level during consultations.	Deepening and Supplementation. The qualitative study provided a micro-level deepening of the ‘medical stress’ dimension, pointing out that the quality and details of clinician-patient interaction​ are a key component shaping the medical stress experience, affecting patients’ psychological feelings.

## 4 Discussion

This study found that the mean total stressor score for patients with breast cancer undergoing postoperative chemotherapy was at a moderate-high level. Combined with the LPA results, there was significant heterogeneity within the patient population, manifesting as four distinct stress profiles: ‘Low-stress Adaptive’ (26.3%), ‘Moderate-high Physio-psychological Distress’ (38.4%), ‘High Medical Concerns’ (20.3%), and ‘High-stress Across Domains’ (15.1%). Notably, over 70% (73.7%) of patients belonged to the latter three moderate-to-high stress profiles, indicating that a substantial majority experienced considerable stress during treatment. The qualitative findings strongly corroborated and deepened this quantitative discovery. In the interviews, all patients described multidimensional and interwoven stress experiences, stemming not only from the physiological suffering of treatment itself (e.g., body image changes, side effects) but, more profoundly, from the comprehensive assault of the disease on their self-identity, family roles, social functioning, and future existential security. The marked variation in the intensity and focus of this stress experience among different patients perfectly aligns with the heterogeneous profiles identified in the quantitative study.

Further analysis indicated that stress experiences were complexly associated with multiple factors. First, disease and treatment burden served as fundamental stressors. In the qualitative interviews, patients described in detail the body image damage, functional decline, and persistent fatigue caused by surgery and chemotherapy, which were highly consistent with the characteristics of the “Moderate-high Physio-psychological Distress” profile. Advanced clinical stage, more treatment cycles, and complications were associated with heightened physical suffering and prognostic uncertainty (20, 21), and a positive association was observed between these factors and patients’ membership in the “High-stress Across Domains” profile. Second, the depletion of psychological and cognitive resources accompanied stronger stress experiences. Patients commonly exhibited profound fear of recurrence and metastasis, decision fatigue in the face of complex medical information, and stigma related to the identity of being a “patient with cancer.” These factors continuously consumed patients’ emotional and cognitive regulatory resources, and particularly for patients in the “High Medical Concerns” and “High-stress Across Domains” profiles, they tended to form a cyclical pattern of “anxiety → resource depletion → increased anxiety.” Furthermore, the buffering or exacerbating role of socioeconomic and support systems was crucial. This study found that younger age and low family income were independent risk factors for the “High-stress Across Domains” profile. Qualitative interviews revealed that financial pressure not only brought direct survival anxiety but also intertwined with feelings of guilt about “burdening the family.” Meanwhile, the quality of patient-clinician interaction (communication details, clarity of information) and the dual nature of family support (serving as both a source of reliance and a potential psychological burden) were closely associated with patients’ perceived stress levels, with particularly notable effects on the “High Medical Concerns” profile. Based on these findings, the following is recommended: 1) Implement tiered interventions:Clinical practice should identify patients’ stress profile characteristics, prioritizing the provision of clear, consistent medical information and decision aids for the ‘High Medical Concerns’ profile to enhance their sense of control; for the ‘High-stress Across Domains’ profile, integrated psychological support, social work involvement, and resource linkage for multidimensional support are needed (22). 2) Build a systematic support network:Incorporate family members into the support system, using family education to help them understand the patient’s psychology and provide non-judgmental emotional support; meanwhile, actively link younger and economically struggling patients to social and financial aid resources to alleviate real-world pressures (23). 3) Addressing stigma and social reintegration: particularly for young patients, peer support groups and counseling on rebuilding social roles during recovery can reduce stigma, enhance social connections and functional recovery, thereby supplementing psychological and social resources and helping to break the cycle of stress (24, 25).

This study identified, through multinomial logistic regression analysis, the key factors associated with patients’ membership in different stressor profiles. These factors were not only supported by quantitative data, but qualitative interviews further elucidated their underlying mechanisms, providing direction for targeted interventions. The study found that older age​ was a significant protective factor. For each one-year increase in age, the risk of belonging to the ‘Moderate-high Physio-psychological Distress’ and ‘High-stress Across Domains’ profiles decreased by 6% and 9%, respectively. Qualitative research provided a vivid explanation for this statistical association: Compared to older patients, younger patients (e.g., P6, P8, P11) are often at a critical stage of career development, family formation, or child-rearing. The social role disruption, sharp income reduction, and worries about family future caused by the disease subject them to a “developmental stress” that surpasses the illness itself. One young patient (P8) admitted: “The child is still young, the mortgage needs paying, my job might be lost. It feels like everything is piling up, with no end in sight.”This multiple layer of stress is highly likely to deplete one’s psychological resources and is associated with a state of complete breakdown. Therefore, it is recommended that clinical staff mark young patients (especially ≤45 years) for stress risk and prioritize them for psychosocial needs screening.​ Interventions should focus on “role re-adaptation” and “future planning,” for example, linking them to career counseling and financial aid resources through social work services; establishing peer support groups for young patients to help them re-anchor short-term life goals within the context of illness, alleviating the sense of loss of control and meaninglessness from interrupted developmental tasks (26).

Economic status is an important factor associated with stress experiences. This study showed that compared to patients with a monthly family income >¥10,000, those with an income ≤¥5,000 had a 3.5 times higher risk of belonging to the ‘High-stress Across Domains’ profile. Qualitative interviews profoundly revealed that financial pressure extends far beyond the numbers of treatment costs; it is deeply bound with feelings of “guilt” and “being a family burden.” Low-income patients (e.g., P1, P11) frequently expressed guilt over depleting family savings and self-devaluation from being unable to fulfill financial duties. This dual “economic-emotional” pressure formed a mutually reinforcing relationship. Therefore, integrating brief screening for financial toxicity into routine care is essential.​ For high-risk patients identified, active support should be provided, including: 1) Informational support:​ Clearly explaining health insurance reimbursement policies, etc., to reduce anxiety from information asymmetry; 2) Resource linkage:​ Assisting by medical social workers in applying for relevant social assistance; 3) Family intervention:​ Guiding family members towards open, non-judgmental communication, helping them understand the patient’s psychological burden, and preventing financial discussions from turning into emotional blame, thereby transforming the family into an effective “pressure reducer” rather than a “pressure amplifier” (27, 28).

Disease severity is directly associated with patients’ stress experiences. The analysis showed that compared to patients with Stage I–II disease, those with Stage III disease had 2.2 and 4.8 times the risk of belonging to the ‘High Medical Concerns’ and ‘High-stress Across Domains’ profiles, respectively. Qualitative data indicate that advanced stage implies a stronger perception of survival threat, more complex treatment regimens, and more uncertain prognosis. Such patients (e.g., P6, P8, P9) are extremely sensitive to medical information; any subtle change in condition or vague clinician-patient communication can trigger intense anxiety. For instance, P4 stated: “If doctors’ explanations differ slightly, I feel unsure and keep wondering if there’s a better option they haven’t told me about.”This suggests that medical care for patients with advanced-stage disease must place extraordinary emphasis on”communication-focused care “alongside providing excellent treatment. Recommendations include: 1) Providing structured disease and treatment plan explanations by the attending physician or specialist nurse to ensure clear, consistent information and enhance the patient’s sense of control; 2) Encouraging patient and family participation in treatment decision discussions and providing decision aids to alleviate “decision fatigue”; 3) Regularly assessing their psychosocial needs and integrating psychological support (e.g., mindfulness-based stress reduction, cognitive-behavioral therapy) as a standard component of comprehensive treatment (29).

The presence of treatment-related complications is associated with stronger stress experiences in patients. This study found that patients with complications had a 2.8 times higher risk of belonging to the ‘High-stress Across Domains’ profile compared to those without. In qualitative interviews, complications (e.g., severe vomiting, peripheral neuropathy, recurrent infections) were described as “the last straw that breaks the camel’s back.” They not only bring additional physical suffering but constantly remind patients of the disease’s tenacity and treatment’s hardship, rapidly depleting their already limited tolerance and regulatory resources. Patient P11 described: “Complications come one after another; as soon as I get through one, another comes. It feels like a constant struggle, with no chance to catch my breath. My willpower is completely worn out. “Consequently, clinical management must attach high importance to the prevention, early identification, and active management of complications, viewing them as a crucial warning signal for psychological crisis. Specifically: 1) Strengthen pre-treatment education, giving patients realistic expectations of potential complications and basic self-management skills; 2) Establish accessible channels for reporting and seeking support for complications, ensuring patients receive timely, effective medical responses when discomfort arises, avoiding amplified anxiety from feelings of helplessness; 3) For patients with existing complications, concurrently assess their level of psychological distress, combining symptom management education with psychological support to help break the vicious cycle of “physical discomfort-emotional breakdown-heightened pain perception” (30, 31). The qualitative analysis of this study also revealed an important yet easily overlooked variable—disease-related stigma. Subtheme 2.3​ showed that younger patients with breast cancer commonly experienced intense feelings of shame and stigma during postoperative chemotherapy, and this feeling was further associated with their active avoidance of social activities. However, the quantitative scale we used (SBSCS) did not specifically measure the dimension of stigma; therefore, the quantitative results may have underestimated the independent contribution of stigma to patients’ psychological stress. Future research should incorporate stigma as a key mediating variable or predictor into the quantitative analytical framework to systematically evaluate its mechanism of action in the pathway of stress formation, thereby achieving a more comprehensive understanding of the complex sources of psychosocial stress in patients with breast cancer and providing evidence-based support for the development of targeted destigmatization intervention strategies (e.g., cognitive restructuring, peer support, social advocacy).Furthermore, determining the optimal screening timing within real-world clinical workflows is critical for effectively implementing tiered interventions. Considering that the chemotherapy process itself continuously influences patients’ stress perception levels, we recommend scheduling the initial screening based on latent profile analysis during the window after surgery but before the first cycle of chemotherapy. At this point, patients have just undergone surgical trauma and are about to face the uncertainties of chemotherapy; stressors are most concentrated and have not yet been confounded by chemotherapy side effects, allowing for a relatively authentic reflection of their baseline psychosocial needs. Subsequent dynamic assessments can be conducted between every two cycles of chemotherapy (i.e., the inter-cycle interval) to capture possible shifts in stress profiles resulting from cumulative toxicity, changes in symptom burden, or feedback on treatment efficacy. This strategy of “baseline assessment before chemotherapy + dynamic monitoring during chemotherapy intervals” balances clinical feasibility with the ability to provide data support for timely adjustment of supportive care plans.

This study has several limitations. First, the cross-sectional design precludes causal inference, and the single-center sample may limit the representativeness and generalizability of the findings. Second, stress assessment relied primarily on self-report scales. Although supplemented by qualitative interviews, the depth of coverage within each profile remains limited, and some nuances of stress experiences may not have been fully captured. Additionally, several potentially important psychosocial variables (e.g., psychological resilience, spiritual well-being) were not included in the analysis. Future studies should adopt longitudinal, multi-center designs combined with multi-dimensional assessment methods to further validate and extend the present findings.

At the methodological level, the sample size used for latent profile analysis was relatively small (n=232), which may increase the risk of class misspecification, particularly in a heterogeneous sample. Although the study strictly followed standard modeling procedures, comprehensively employing multiple fit indices (AIC, BIC, aBIC, entropy, LMR-LRT, and BLRT) to determine the optimal number of classes and ensuring the clinical interpretability of each profile, the modest sample size may still result in the omission or merging of low-probability profiles. Moreover, there is inherent uncertainty in model selection: although the four-profile model performed best across fit indices, the three-profile model also demonstrated statistical plausibility with more balanced class sizes. In the four-profile model, the “High-stress Across Domains” profile comprised only 15.1% of the sample (n=35); thus, further analyses involving this subgroup should be interpreted with caution. Future research should replicate the four-profile structure in larger, more representative samples and explore whether the three-profile model may be more practically useful in certain clinical contexts. The qualitative sample was also limited in size (n=12), which may raise concerns about the representativeness of the qualitative findings and theoretical saturation. Of note, purposive maximum variation sampling was employed to ensure coverage of patients across different stress profiles, and the principle of information saturation was followed during data analysis—recruitment ceased when new interviews no longer generated novel themes or concepts. Nevertheless, the limited sample size may still have missed certain rare but important subgroup experiences. Future studies should conduct multi-center qualitative investigations on a larger scale, combined with longitudinal tracking designs, to further validate and expand upon the present findings. Regarding variable selection, two shortcomings should be acknowledged. First, menopausal status was not included in the analysis. Previous evidence (32) suggests that menopausal status may influence the intensity and pattern of stress responses in breast cancer patients, with differences in stress-related associations between premenopausal and postmenopausal women. As menopausal status was not systematically collected in this study, we were unable to control for this potential confounder or examine its association with stress profile membership. Second, age was treated as a continuous variable. While this approach preserves informational integrity, it may obscure non-linear variations in stress experiences across different age groups. Previous studies have often used 45 or 50 years as age cutoffs for breast cancer patients to distinguish pre- and postmenopausal status and differing psychosocial needs. Future research should incorporate menopausal status as an important biological and psychosocial variable, explore optimal age cutoffs, and test non-linear relationships between age groups and stress profiles to provide more clinically actionable stratification criteria.

## 5 Conclusion

This study employed an explanatory sequential mixed-methods design to gain in-depth insight into the heterogeneity of stress experiences, their influencing factors, and the underlying formation mechanisms among patients with breast cancer undergoing postoperative chemotherapy. The findings indicate that the stress experiences of these patients are not homogeneous; rather, they manifest in four stable and distinct heterogeneous patterns. Over 70% of patients experienced moderate to high levels of stress, confirming that psychosocial stress is a significant and widespread, yet variably expressed, challenge for this population. Secondly, the study identified key risk and protective factors determining profile membership. Advanced age emerged as a significant protective factor, whereas younger age, low monthly family income, advanced clinical stage, and the presence of treatment-related complications constitute a core cluster of risk factors for belonging to the ‘High-stress Across Domains’ profile, the highest-risk category. The qualitative findings and the quantitative results mutually validated, deepened, and complemented each other, jointly revealing the dynamic psychosocial mechanisms underlying the formation of different stress profiles. The practical significance of this study lies in providing direct and specific targets for developing and implementing tiered and precise psychosocial interventions.

## Data Availability

The original contributions presented in the study are included in the article/**Supplementary Material**. Further inquiries can be directed to the corresponding author.
