# Supplementary material for: Analysis of latent profiles of stressors and their influencing factors in patients with breast cancer undergoing postoperative chemotherapy: a mixed-methods study
Source: Front Oncol. 2026 Aug 21;16:1918956. doi: 10.3389/fonc.2026.1918956 (PMC13541498; doi:10.3389/fonc.2026.1918956)
Supplement: Supplementary file 1 [file DataSheet1.docx]

**Supplementary Figure 1**

**Profile 1 (Low-stress Adaptive, 28.0%) scored lowest across all five dimensions. Profile 2 (Moderate-high Physio-psychological Distress, 41.4%) showed elevated scores on Physical and Sexual Stress, Interpersonal Stress, and Worry and Concern about the Future. Profile 3 (High-stress, 30.6%) was formed by a weighted merger of the four-profile model's "High-stress Across Domains" (15.1%) and "High Medical Concerns" (20.3%) profiles, with particularly high scores on Medical Care Stress and Worry and Concern about the Future.**

**Supplementary Figure 1. Standardized scores of the three-profile model for the five stressor dimensions of the SBSCS among patients with breast cancer undergoing postoperative chemotherapy (N=232).**
